# BISR-RNAseq: an efficient and scalable RNAseq analysis workflow with interactive report generation

**DOI:** 10.1186/s12859-019-3251-1

**Published:** 2019-12-20

**Authors:** Venkat Sundar Gadepalli, Hatice Gulcin Ozer, Ayse Selen Yilmaz, Maciej Pietrzak, Amy Webb

**Affiliations:** 1 Biomedical Informatics, The Ohio State University, Columbus, OH USA; 2 The James Comprehensive Cancer Center, The Ohio State University, Columbus, OH USA; 3 Bioinformatics Shared Resource Group, The Ohio State University, Columbus, OH USA

**Keywords:** RNAseq, Transcriptome, Workflow, Visualization

## Abstract

**Background:**

RNA sequencing has become an increasingly affordable way to profile gene expression patterns. Here we introduce a workflow implementing several open-source softwares that can be run on a high performance computing environment.

**Results:**

Developed as a tool by the Bioinformatics Shared Resource Group (BISR) at the Ohio State University, we have applied the pipeline to a few publicly available RNAseq datasets downloaded from GEO in order to demonstrate the feasibility of this workflow. Source code is available here: workflow: https://code.bmi.osumc.edu/gadepalli.3/BISR-RNAseq-ICIBM2019 and shiny: https://code.bmi.osumc.edu/gadepalli.3/BISR_RNASeq_ICIBM19. Example dataset is demonstrated here: https://dataportal.bmi.osumc.edu/RNA_Seq/.

**Conclusion:**

The workflow allows for the analysis (alignment, QC, gene-wise counts generation) of raw RNAseq data and seamless integration of quality analysis and differential expression results into a configurable R shiny web application.

## Background

A whole transcriptome sequence provides an estimate of the quantity of all transcripts present in a group of cells. High throughput sequencing technologies have been developed to deep sequence the transcriptome. Sequencing generates several million short reads that are typically 50–400 bases in length. These reads can be mapped to a known reference genome or assembled de-novo. Either method will provide a snapshot of the transcript present in the sample and an estimate of abundance. Statistical methods have been developed to normalize and compare transcript estimates to identify differential transcripts. At each step of the bioinformatics analysis pipeline, there are many options for specific programs to use, reference genome for alignment, and gene annotation set of expression quantification. One of the challenges for the analysis of transcriptome data is to have a reproducible set of steps for consistent analysis. The aim of this study was to generate a standardized workflow available to the public that would make RNAseq analysis easier to implement, especially for non-expert users.

The growth of genomics data has been exponential over past 5 years. The workflow established by various researchers to store, analyze and deliver the results have been scaling in order to meet the requirements of large scale data. Open source software and technology have been widely adapted to address the requirements in genomics data analysis. Interpreting, understanding and communicating the results in genomics is commonly done using respective plots and tables from the data analysis outputs. There are many open source software such as R [1], Bioconductor [2], Shiny [3] that have facilitated researchers to explore insights in genomics data. However, leveraging these open source technologies in a scalable way is still a challenge for analysts or users who are not familiar with these open source technologies. As the Bioinformatics Shared Resource (BISR) group at OSU, we developed this workflow to provide consistent analysis and reports to our collaborators. Other groups have developed workflows and pipelines to streamline RNAseq analysis [4–7]. Unlike other applications, our approach is easily scalable as it can run multiple samples in parallel in a pbs scripting environment. It allows us to retain version control with a config file and pbs script detailing particular options and versions used in the workflow. This allows an expert user to switch version s or programs of the workflow including alignment program or reference genome. The shiny application offers interactive visualizations and has an R backend that takes advantage of R packages typically used in RNAseq analysis. Through an interaction with OSC, we have a storage environment for shiny reports providing protected, private access for end users.

Using Shiny and other libraries, we designed a scalable workflow for creating interactive RNAseq reports which can be easily launched and customized by users who are not expert users of shiny or R. Our workflow is one of the first to pipe into a customizable R shiny application for visualization and reports generation.

## Implementation

Example datasets can be downloaded from GEO repositories in fastq format using ‘fastq-dump’ from NCBI.

If reference files are not available, gather required files: download reference genome and index for hisat2 use, download bed files from RSeQC for gene definitions and ribosomal gene locations, download gene annotation file appropriate for genome. Install all used programs and have locations included in your PATH.

Clone RNAseq_pipeline from github (https://github.com/MPiet11/BISR-RNAseq)

Generate a sample file containing 3 columns—name of forward read fastq, name of reverse read fastq, and name of sample. Sample name should not contain any dashes. For single end datasets, leave NA as a placeholder for the reverse read. Scripts assume fastq files will be in a folder within the working directory named “fastq.” Fastq can be gzipped or as is. If gzipped, name of fastq in sample file should reflect this.

Edit config file with full path and names of required parameters (sample file, pbs script, reference genome, gene annotation, ribosomal bed, and gene definition bed).

Execution of the config file will pass the entered parameters to a shell script which will submit a job for each sample to run the provided pbs script. Modify pbs header for resources provided by your specific pbs computing environment.

Provided pbs script will execute the following pipeline:

1. Raw read QC with fastqc for forward and reverse read fastq. Results from different samples can be gathered with multiQC
2. Raw read alignment with HISAT2
3. Convert sam to bam, sort, and index with samtools
4. Generate post alignment QC metrics with RSeQC (detected junctions, read distribution, experiment type, and ribosomal contamination) and picard (insert size and duplication rate)
5. Count reads per gene with featureCounts

After all samples have finished, provided script ‘rnaseq_final_reports.sh’ can be submitted to gather QC and counts. This script requires datamash for table reformatting. Script will also gather unnecessary files into a trash folder. Finally the script runs 2 provided R scripts—1. ‘read_data_for_rshiny.R’ will read all QC/counts tables into an rds object. 2. ‘create_rshiny_input.R’ will run differential expression (set up in DGE_RNAseq_limma.R) calculating a pair-wise comparison of groups using limma and package all results into an rds object for shiny. Differential expression requires a file with gene_ID, gene names, and biotype and a file with sample names and groups for comparison. Place these files in a folder named ‘raw_data’.

Detailed installation instructions are provided in the README file on the project gitlab https://code.bmi.osumc.edu/gadepalli.3/BISR_RNASeq_ICIBM19. Overall the steps are as follows

1. To run the shiny app clone the git repository to local computer or a server that runs shiny.
2. The input files for R shiny report should be transferred into the ‘data’ folder under the shiny app folder. The app currently is setup with packrat package manager, but the choice is left to the user to discard it and install packages as required.
3. It is important to make sure that the R libraries are loaded as required. For this purpose, the user can run the load_project_packages.R in R IDE or using a command line ‘Rscript load_project_packages.R’. This script loads the library if it is already present or it will install the missing libraries.
4. Finally, the user would need to make sure that names of the input files match to those listed under read_data.R. If there is a naming difference, these should be fixed in order for the shiny app to read the data.
5. To launch the app the user can run ‘shiny::runApp(‘app. R’)’.

To launch the shiny app over a webserver it is required to install and setup R shiny server. The installation and setup instructions are detailed in the Rstudio help pages https://www.rstudio.com/products/shiny/shiny-server/. The setup of the app to run with user data follows the above same steps.

## Results

**Fig. 1:**
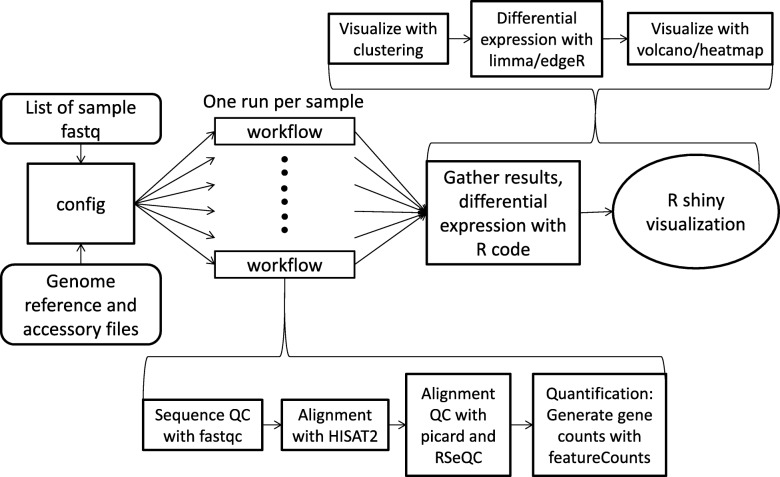
Schematic workflow of RNAseq pipeline. A list of fastq files and locations of necessary reference files are fed into config file which spawns a workflow run job for each sample. Results are gathered into an R data object and differential expression is calculated through provided R code. Visualization is provided through R shiny app

A conceptual outline of this workflow is presented in Fig. 1. A set of fastq formatted sequencing files are fed into the parallel alignment and gene counts generation is shown in the workflow. For a given dataset, we make a tab-delimited three column sample manifest listing the forward read, the reverse read, and a name that will be used to identify the sample through analysis. If a sample is run on multiple lanes, we recommend leaving them separate so that QC can be assessed on individual lanes. Counts from different lanes can be summed at the end. For the purposes of this article, raw data was downloaded from NCBI’s Gene Expression Omnibus (GSE48403 [8]). Scripts are setup to be run in a high performance computing (HPC) environment that utilizes portable batch system (PBS) for job scheduling and could be easily modified for other schedulers such as slurm.

Run time variables and paths are set in the config file. This includes reference genome, gene annotation gtf, bed files needed for RSeQC [9], name of pbs script, etc. The pbs script runs a pipeline with default setup preferred by our group. Advanced users can modify this pbs script with additional program options or switch out the programs used for particular steps (for instance run STAR [10] instead of HISAT2 [11]). We assume all required programs have been installed and accessible on the cluster environment. Locations for python applications like RSeQC [9] will need to be added to the PYTHONPATH. The workflow also assumes that you have generated reference genome indexes appropriate for your alignment program and that you have a gene definition file in gtf format in the same coordinates as your genome.

A sample file is a tab-delimited 3 column file generated by the user listing the forward and reverse fastq files and a name for the sample. Executing the config file launches a workflow job for each sample listed in the sample file and runs through the set of steps laid out in the pbs script.

The first step in the analysis of RNAseq raw data is to assess the QC of the raw sequence. We accomplish this by generating a view of the data in FastQC and summarizing those views with mutliQC [12]. multiQC generates an html document summarizing all FastQC results across multiple samples. Important checks include sequence quality along the length of the read and adapter content. Any adapter content can be trimmed with Cutadapt [13] or Trimmomatic [14] but the workflow here assumes adapters are not present.

Raw fastq files are aligned using HISAT2 [9] with default options to the reference genome specified in the config file. For alignment we used Ensembl’s GRCh38 [15] reference genome indexed using hisat2-build. HISAT2 generates summary statistics on overall mapping rate and uniqueness of mapping.

Alignment QC is generated using RSeQC [7] and picard [16] to assess: duplication rate, insert size for paired end reads, ribosomal contamination, proportion of known/novel junctions detected, read distribution across genomic features, library preparation approach based on paired read alignment. Gene definition and ribosomal gene location in bed format were downloaded from the RSeQC website. The main aim of the alignment QC is to check whether the alignment is consistent across samples and that it confirms what is known about the library preparation.

We use featureCounts [17] from the subread package to count reads on genes. In-house we prefer using the primary option for multimapped reads. Gene annotation definition file in gtf format is specified in the config file. For this example, we used Ensembl gene annotation release 92 [18].

R scripts are provided to gather QC metrics into an rdata object, run simple pairwise differential expression, and format the data for display by the R shiny app. Expert users with a more complicated experimental design could write their own code for differential expression.

**Fig. 2:**
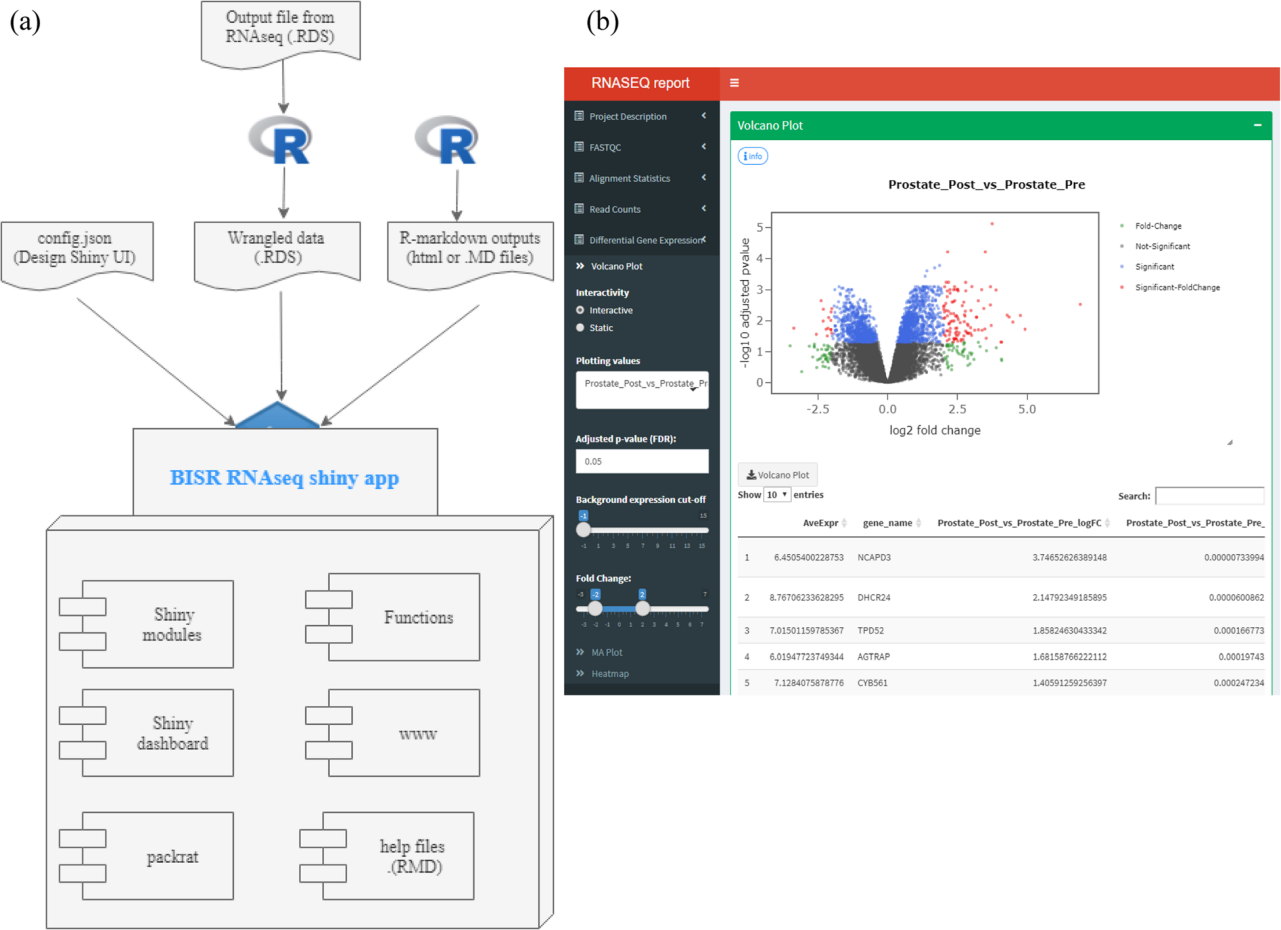
The overall design of the BISR RNASeq shiny app. **a** Data gathering: The 3 inputs files that BISR shiny app takes as inputs (1) config.json file, that defines the shiny UI (2) a .Rds object generated by custom R script run on RNAseq pipeline output (3) files relevant to the project that are generated as Rmarkdown or html files. These three items are sent into the app which is made up of the following components **b** A screen shot of BISR RNAseq report

Wrapping sequencing data analysis into an interactive framework enhances the exploration of large scale genomics data effortlessly. Shiny is a web application framework [3] that facilitates R users to build interactive visualizations on the data analysis outputs However, to implement it at analysis core facility would need to build a production level software. The setup presented here allows seamless integration with experimental designs that can be easily customizable without the expertise in web programming. To achieve this task, we have designed and developed interactive reports in shiny that offer generality and extensibility. The overall design is detailed in Fig. 2. Figure 2a, outlines the different inputs for BISR shiny app. A configuration file, wrangled data object, and project relevant accessory files. The configuration file in JSON format stores the information on what user interface (UI) components should the shiny app render. The goal of the configuration file is to allow non-shiny and R users ability to customize their UI and launch their data analysis findings. A complete configuration file is provided the source code under the ‘data’ folder. This allows a non-shiny user to customize or change UI components. The wrangled data in RDS format stores the information about the specific data values and parameters for respective plots and tables to be displayed on the UI. Finally, the project detail files comprise of any html or Rmarkdown files that provide relevant information for respective RNA sequencing project. These project detail files are optional, but to run the app it is required to provide the JSON configuration file and as well as the data. RDS object.

The Fig. 2b, show an overview of the components of the BISR shiny app. These components comprises of specific codes that offers extensibility and scalability. In order create a self-contained app we used packrat [19], an R-library manager that offers seamless deployment of apps across different operating systems. The interactive shiny report that BISR delivers comprises of sequence quality analysis plots, differential gene analysis plots and their respective tables. To reduce the repeatability of code and enhance the reproducibility we developed shiny modules for respective plots. These small composable shiny modules are extensible across different apps. CRAN and Bioconductor libraries were employed to achieve this task.

Figure 2b displays the output in the RNAseq report. The left hand panel shows the different subsections provided in a report as part of the configuration JSON file —Project description, QC metrics, read count table, and differential expression results. An example figure is provided in Additional file 1 and the complete JSON file is included in the source code under the ‘data’ folder. Differential expression results are presented as a volcano plot, MA plot, and heatmap. Each plot is customizable based on FDR, fold change, and base line expression levels. The customized results can be exported for publication or further analysis.

## Discussion

This workflow was created to address issues encountered when processing a large number of RNAseq datasets. First, we wanted a workflow that would run sequence of commands in a consistent manner. Second, we wanted to keep a record of runtime details including: genome version, gene annotation set, program version, program options, etc. Third, we wanted a smooth transition from generating RNAseq results from the workflow to visualization through a shiny app.

This workflow encourages consistency between RNAseq analysis datasets. The workflow is intended to be downloaded as a self-contained directory where the user can add their own fastq files and sample file. The first step is to run the config file to generate QC, alignment, and counts. As technologies and software improves, programs called in the bulk of the workflow could be switched out with minimal effort as long as users are conscious about with the program needs and what is generated as an output. The second step is to gather QC and counts, compute differential expression, and package the results for our R shiny app. The third step is to launch the shiny app and view the results. The shiny app provides a user-friendly visualization for the data, understandable for even non-bioinformatics users. If raw data comes in batches, it is easy to reuse a set config file and pbs script.

Most of the workflow parameters are set in the config file or the pbs script. Retaining these files will allow a user to keep a record of run-time details such as program versions and genome versions. With these details and the raw fastq, the analysis could be recreated at any point in the future.

This application provides a smooth transition from QC and counts generation to visualization. The provided R scripts pull results from individual samples into summary tables and calculate differential expression. All results are stored in an. Rds object with additional details needed for R shiny visualization including parameters that are needed for respective plots. A large benefit to the R shiny application environment is automatic plotting of interactive graphs. Graphs for QC are plotted with options to scale based on library size or unscaled. Publication ready plots can be exported as .png and the filtered and unfiltered tables can be downloaded in CSV format. Differential expression results are visualized as volcano plots, MA plots, and heatmaps and all plots can be filtered for FDR, fold change, and expression levels.

The goal of this project is to provide a scalable and configurable RNAseq pipeline that can run the analysis and as well as integrate the results in a seamless way. Bioinformaticians who are not familiar with shiny can easily customize the UI end of the shiny so that creating project specific interactive report is effortless. As noted in the Fig. 2b, the sidebar contents (controllers and input boxes) and the body contents (plots and tables) for the Volcano plot are determined by the config. JSON. The Fig. 2b, displays a Volcano plot and has certain controllers to interact with the data displayed on the plot. While the JSON file offers customization to at menu, submenu and box levels. When the name, tabname (id), data frame name, display and UI and Server module are changed the UI renders accordingly.

## Conclusions

Presented is a consistent workflow to analyze RNAseq data and generate interactive reports and customizable visualization suitable for publication. The application is simple to use and set up to parallelize analysis in a supercomputer environment. This workflow empowers researchers without bioinformatics or programming experience to run quick analysis on their data by working with human readable configuration files. The user-interface on the shiny end is configurable with a simple JSON file thus facilitating generation of interactive visualization report across different RNAseq projects. The resulting R shiny report and figures are suitable for non-bioinformatics use and for guiding future biological research. Work-in-progress to create an R package and an active development process to implement feasible feature requests from users on github.

## Supplementary information

**Additional file 1.** Example of JSON file customization translated into R shiny display.

## Data Availability

Project name: BISR-RNAseq Project home page: workflow: https://github.com/MPiet11/BISR-RNAseq and shiny: https://code.bmi.osumc.edu/gadepalli.3/BISR_RNASeq_ICIBM19 Operating system(s): RNAseq pipeline workflow: Linux. Shiny workflow: Windows, Linux/shiny server Programming language: BASH, R, python Other requirements: RNAseq pipeline workflow: Installed analysis programs (fastqc, hisat2, samtools, rseqc, picard, featureCounts), reference genome and gene annotation gtf. For shiny, Rstudio IDE (suggested). All the packages required to launch interactive report are listed under ‘load_project_packages.R’. License: Open Source (MIT) Any restrictions to use by non-academics: none

## Abbreviations

BISR: Bioinformatics shared resource
GEO: Gene expression omnibus
HPC: High peformance computing
IDE: Integrated development environment
NCBI: National Center for Biotechnology Information
OSU: Ohio State University
PBS: Portable batch system
QC: Quality control
RNA: Ribonucleic acid
UI: User interface
