# Supplementary material for: BISR-RNAseq: an efficient and scalable RNAseq analysis workflow with interactive report generation
Source: BMC Bioinformatics. 2019 Dec 20;20(Suppl 24):670. doi: 10.1186/s12859-019-3251-1 (PMC6923898; doi:10.1186/s12859-019-3251-1)

# Supplementary figure


```
{
  "section_1": {▭
  "section_2": {▭
  "section_3": {▭
  "section_4": {▭
  "section_5": {
    "Menu": [{
      "name": "Differential Gene Expression",
      "tabName": "menuitem5",
      "display": 1
    }],
    "Submenu": [{
        "name": "Volcano Plot",
        "tabName": "menu5subitem1",
        "display": 1,
        "Uimodule": ["VolcanoInputUI"],
        "data": "volcanto_plot",
        "Servermodule": ["VolcanoServer"],
        "box": [
          {
            "id": "menu5subitem1box1",
            "title": "Volcano Plot",
            "Uibody": ["VolcanoPlotUI"],
            "display": 1
          }
        ]
      },
      {▭
      {▭
    ]
  }
}
```


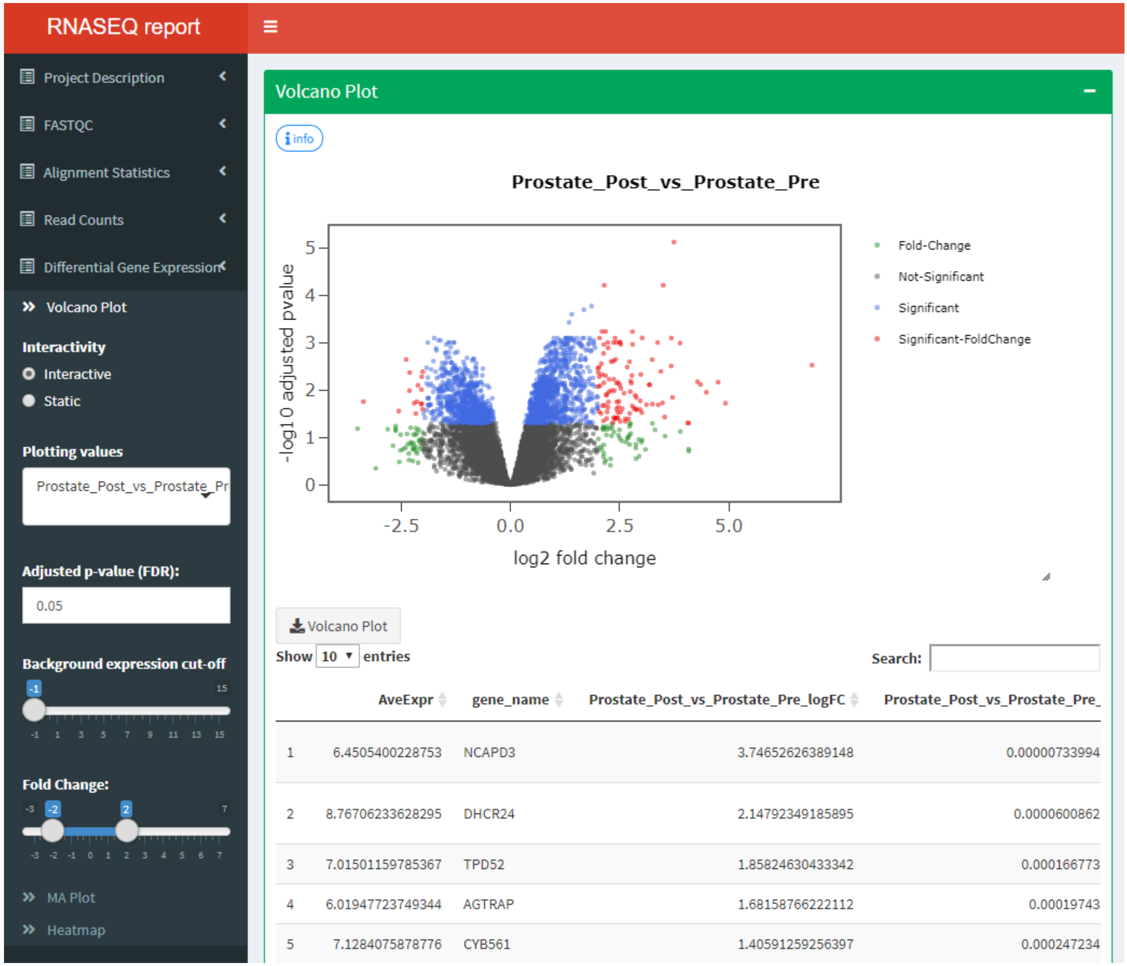

Supplement: Supplementary file 1 — Additional file 1. Example of JSON file customization translated into R shiny display. [file 12859_2019_3251_MOESM1_ESM.pdf]
